# From non-tidal to tidal environments: movement behaviour of Chinese mitten crabs on downstream spawning migration

**DOI:** 10.1186/s40462-025-00548-3

**Published:** 2025-04-03

**Authors:** Heleen Keirsebelik, Pieterjan Verhelst, Bram D’hondt, Jonas Schoelynck

**Affiliations:** 1 Department of Biology, ECOSPHERE Research Group, University of Antwerp, Universiteitsplein 1C, 2610 Wilrijk, Belgium; 2 Research Institute for Nature and Forest (INBO), Havenlaan 88, Box 73, 1000 Brussels, Belgium

**Keywords:** Crustacea, Decapod, Circadian patterns, Tidal patterns, *Eriocheir sinensis*, Acoustic telemetry, Scheldt Estuary

## Abstract

**Background:**

The Chinese mitten crab (*Eriocheir sinensis*) is a widespread species that is both threatened and commercially valuable in its native range, but considered invasive in various other parts of the world. Being catadromous, their downstream spawning migration to the sea marks the crucial final step in their life. Yet, little is known about their behaviour during this migration.

**Methods:**

In this study we investigated the migration of mitten crabs from non-tidal freshwater rivers to the tidal estuarine mouth over a distance of 125 km using acoustic telemetry. During a three-year period, a total of 34 adult mitten crabs were equipped with acoustic tags. Six were equipped with tags that also had an accelerometer and pressure sensor to record the activity and depth of the crabs.

**Results:**

All mitten crabs migrated downstream, primarily residing within the deeper parts of the rivers. They were detected until the border between the mesohaline and polyhaline zone of the estuary, suggesting that this area serves as their spawning habitat. Migration speeds were significantly higher in non-tidal freshwater rivers (on average 4.65 ± 3.51 km day^−1^, range: 0.06–15.37 km day^−1^) compared to the tidal estuary (on average 1.29 ± 1.22 km day^−1^, range: 0.05–8.19 km day^−1^). Mitten crabs migrated primarily during the darker hours of the day, however this pattern diminished in the estuary. In tidal rivers migratory activity was largely driven by the tidal cycle, with crabs selectively moving downstream during the ebb tide. No behavioural differences between male and female crabs were observed.

**Conclusions:**

During their spawning migration, adult mitten crabs reveal movement behaviour that maximises their fitness. In shallow non-tidal rivers, migrating at night likely reduces predation risk. In tidal rivers, this behaviour largely disappears, which could be linked to increased depth and turbidity, or the prevalence of the tidal migration cue. Based on detection and acceleration data, this study provides the first evidence that adult mitten crabs use selective tidal stream transport during their migration. As a slow-moving species, this behaviour helps to preserve energy for spawning during the challenging final phase of their life cycle.

**Supplementary Information:**

The online version contains supplementary material available at 10.1186/s40462-025-00548-3.

## Background

While most brachyuran crabs (Crustacea: Decapoda) spend their entire lives in marine environments, primary freshwater crabs complete their full life cycle in freshwater or terrestrial habitats [68]. A distinct group, known as secondary freshwater crabs, are fully adapted to freshwater or terrestrial environments but still require the marine environment at some stage of their life cycle [35]. These crabs often undergo seasonal migrations, sometimes covering great distances to reproduce [8, 26, 27, 56, 59].

One well-known example of a crab species that undertakes long-distance spawning migrations from fresh to salt water, is the catadromous Chinese mitten crab (*Eriocheir sinensis*, H. Milne Edwards 1853). The Chinese mitten crab is native to the coastal regions of the Yellow Sea, including northern and central China, and Korea. However, the species has a global distribution as a result of multiple successful invasions following (accidental) human introductions through e.g. ballast water or escapes from markets [15]. Within its native range, the species is commercially valuable, but under threat due to overfishing, habitat degradation and obstructed migration routes [9, 33, 70]. Conversely, in its non-native range the species can be highly abundant, as evidenced by large numbers in northwestern Europe, and especially Belgium [16, 58]. Here, the species is managed because of concerns about its ecological impact through e.g. predation on native species, damaging of macrophytes and destabilizing river banks through burrowing [22, 51, 52, 58, 64].

The species’ life cycle starts in saline waters at sea or in estuaries, where the larvae hatch from eggs and develop through five stages into a megalopa larva and subsequent juvenile crab [5, 36]. These juvenile crabs move into the estuary and generally reside in brackish to freshwater tidal areas [22, 53]. In spring, the juvenile crabs migrate upstream and swarm out, searching for suitable habitat up to hundreds of kilometres inland [47, 54]. Once they reach maturity, aged one to five years, the adult crabs venture back seaward to reproduce [15]. Typically, male adult crabs start their spawning migration in early autumn, followed by the female crabs [47]. After mating in brackish water, the female crabs carry the fertilized eggs to more saline water. One brood can contain up to a million eggs [47–49].

The timing of their downstream migration appears to be consistent across both their native and non-native range, starting in late August or September and lasting until December [55]. Conversely, the timing of mating, brooding and larval release differs between regions [15]. Mitten crabs make this spawning migration only once in their lives and perish soon after the eggs hatch [47]. This is a result of the high energetic cost of the migration and reproduction itself [40, 47]. They are exposed to predation and dramatic shifts in environmental conditions [38, 46]. On top, in highly regulated water systems they encounter barriers that can force them to temporally leave the river with the added risk of desiccation [12], and references therein, [17].

Although the general life cycle of the Chinese mitten crab is fairly well understood, little is known about their behaviour during their spawning migration. General migration speeds have been derived from mark-recapture studies and range between 0.2 and 12 km day^−1^ [47, 58]. However, it is not known whether there are differences between sexes, whether they migrate continuously or take breaks, or whether their movement rates and behaviour change across non-tidal and tidal environments. To maximise survival and conserve energy for reproduction, mitten crabs likely adjust their migratory behaviour. For instance, to alleviate the energetic costs of movement, it is assumed that mitten crabs ‘go with the flow’. This is continuous in non-tidal rivers, but the direction of the current changes over time in a tidal environment. In the latter, mitten crabs could use selective tidal stream transport (STST), only migrating during the favourable conditions of the ebbing tide. This energetic-efficient behaviour is common among marine and estuarine animals, such as anadromous and catadromous fish, and has been demonstrated for many larval stages of brachyuran crabs [20, 21]. Additionally, predator avoidance behaviour can obviously increase survival. Therefore, mitten crabs likely migrate primarily at night, which has been suggested based on catch data [53].

Much of our current understanding of the migration and spawning behaviour of mitten crabs has been derived from catch data (e.g. [10, 11, 45, 47, 67]). While valuable, this data offers only a coarse resolution of their behavioural patterns. With the emergence of acoustic telemetry, it is possible to study the movement behaviour of individual aquatic animals and thus to investigate their spatial ecology in detail [32]. A growing body of literature has proven the use of this technique to track decapods [18] and references therein).

In this study, adult Chinese mitten crabs were tracked for the first time using acoustic telemetry. We investigated their behaviour during their downstream spawning migration from upstream freshwater areas to brackish zones near the river mouth. Individual crabs were tracked to (1) Identify the temporal and spatial scale of the migration, (2) Investigate circadian and circatidal patterns in behaviour such as STST, and (3) Assess movement rates. By elucidating their migratory behaviour, this study contributes significantly to the current knowledge of the ecology of an economic and ecologically important species, and by extension, of adult stages of catadromous brachyuran crabs.

## Methods

### Study area

**Fig. 1:**
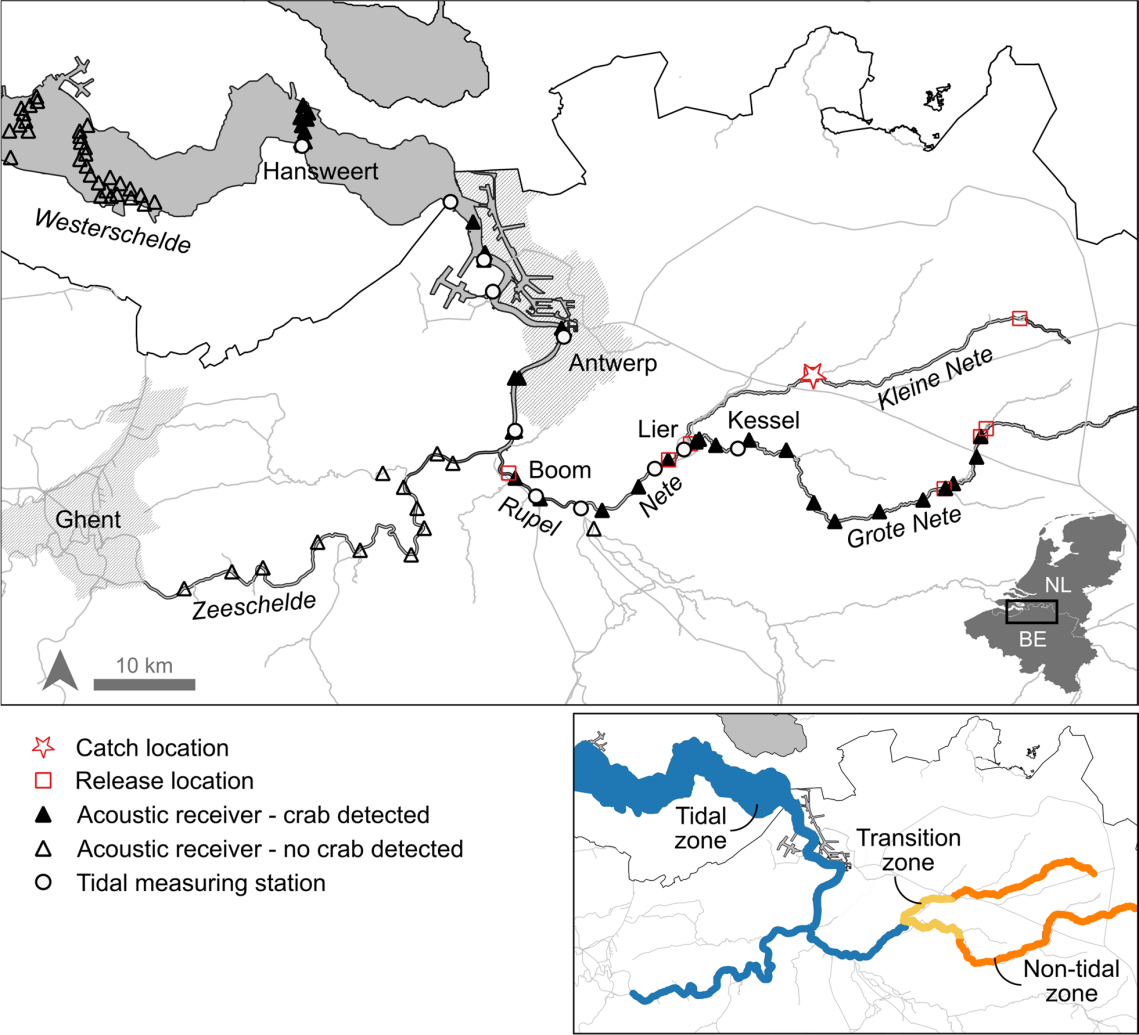
Map of the study area with the names of the rivers and estuary indicated in italics. Solid triangles indicate acoustic receivers where crabs were detected, hollow triangles indicate acoustic receivers in the study area where crabs were not detected. The map below shows the three tidal classes: a tidal zone with meso- to macrotidal influence in blue, a tidal transition zone with microtidal influence in yellow, and non-tidal river sections in orange

The field study was performed in the Scheldt River basin. The Scheldt Estuary is a principal migratory route of the Chinese mitten crab and its many tributaries and surrounding (tidal) areas provide optimal habitats for the crabs’ different life stages. The River Scheldt is a 355-km-long river that flows from Saint-Quentin (northern France) through Belgium to the North Sea near Vlissingen (The Netherlands). The surface area of the Scheldt river basin is approximately 21,863 km^2^. The Scheldt Estuary extends over two different zones: (1) The Westerschelde (58 km, depth 13–50 m) between the Belgian-Dutch border and Vlissingen and (2) The Zeeschelde (105 km, depth 6–20 m) between Ghent and Antwerp (Fig. 1).

Based on tidal influence, the study area can be divided in three classes (Fig. 1). First, a tidal zone (± 125 km) comprising Rivers Nete and Rupel, and the Scheldt Estuary, which are all under strong tidal influence, ranging from meso- (average tidal range between 2 and 4 m) to macro-tidal (average tidal range exceeding 4 m). This tidal zone covers the whole salinity gradient from fresh to salt water. The rivers are rain-fed, leading to highly variable discharge among seasons, with a peak in discharge during winter. Therefore, the transition zone between fresh and salt water shifts throughout the year [44]. Generally, the zone between the river mouth of the Scheldt Estuary and Hansweert (the Netherlands) is polyhaline (> 30 PSU), while the zone between the Belgian-Dutch border and the vicinity of Antwerp is mesohaline (5–18 PSU). The zone from Antwerp until the confluence with the River Rupel, and the River Rupel itself, are oligohaline (0.5–5 PSU). Second, a transition zone (± 11 km) between this tidal zone and unidirectional rivers without tidal influence, comprising the lower reaches of Rivers Grote and Kleine Nete. The lower reaches of these rivers experience limited tidal influence (micro-tidal), with an average tidal range of less than 2 m (Fig. S2, Kessel). This tidal influence is presumably affected by a culvert near their confluence and gradually fades out about 14–17 km upstream. This tidal transition zone is strictly fresh water. Thirdly, a non-tidal zone (± 30 km), comprising the upstream reaches of Rivers Grote and Kleine Nete, which are shallow, rain-fed, freshwater rivers with no tidal influence.

Within the tidal zone, the duration of the tidal cycle is on average (± SD) 12.4 ± 0.4 h, but there is considerable variation in the duration of each tidal phase within the study area (Fig. S2). Ebb and flood are almost symmetrical in the Westerschelde (e.g. Hansweert: 6.3 ± 0.3 h ebb, 6.1 ± 0.2 h flood) and become increasingly asymmetrical upstream. This results in a longer ebb tide and shorter flood tide in the Zeeschelde (e.g. Antwerp: 6.9 ± 0.3 h ebb, 5.5 ± 0.3 h flood) and River Rupel (e.g. Boom: 7.1 ± 0.3 h ebb, 5.3 ± 0.3 h flood) up until the most upstream tidal station of the Nete River around Lier (8.4 ± 0.4 h ebb, 4.0 ± 0.4 h flood).

### Acoustic network

In 2014, the Permanent Belgian Acoustic Receiver Network (PBARN) was set up in the Scheldt river basin and the Belgian part of the North Sea as part of the Belgian LifeWatch project (Fig. 1) [50]. The detection range of the receivers depends on different environmental variables, and ranges between < 300 m and 1005 m [7]. In the Westerschelde, the acoustic receivers (model VR2W, InnovaSea Systems Inc., USA) are deployed on marine navigational buoys in three separate lines over the full width of the river. With a 3–5 m long chain and a 10–17 kg weight at the end, hydrophones are pointed downwards. Due to the dependency on these buoys, a complete detection coverage over the full width of the arrays was not possible. In the Zeeschelde and in Rivers Rupel, Nete and Grote Nete, the acoustic receivers are attached to chains on the river bank, held in place with a 10–17 kg weight and kept upright with a small buoy. In the broader parts of the rivers, receivers are placed on both river banks to cover the full river width. During the period of this study a total of 86 receivers were deployed within the study area (Fig. 1). Note that the receiver network extends further into the Belgian part of the North Sea, but is not shown in Fig. 1 since we did not detect Chinese mitten crabs on that part of the network.

### Tagging procedure

Adult Chinese mitten crabs were caught during their downstream migration in a fixed crab trap in the Kleine Nete River (Fig. 1, see [58] for details on the fixed crab trap) in three consecutive years (2020 till 2022). This trap was used because it allows efficient capture of large numbers of migrating Chinese mitten crabs. The large catch numbers allowed for selection of sufficiently large, size-matched and healthy individuals, while maintaining a balanced sex ratio. Large individuals (carapace width > 55 mm) were selected to ensure that the terminal moult was completed and that these crabs were ready to spawn. Furthermore we checked whether the crabs were ‘healthy’; i.e. active, no limbs missing and no damages to the carapace. The sex, carapace width (to 0.01 mm) and wet weight (to 0.01 g) of the individual crabs were determined (Table S1).

**Fig. 2:**
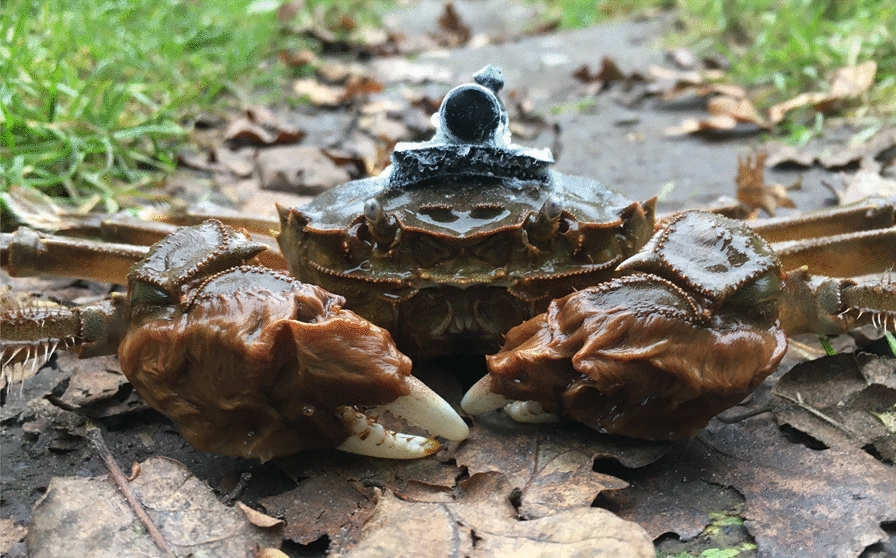
Adult male Chinese mitten crab with acoustic tag prior to release in November 2021

The crabs were tagged in the laboratory of the University of Antwerp. The dorsal part of the carapace was dried with paper towel and sanded superficially with a rotary tool. As such, the carapace becomes roughened, which improves attachment of the tag [13]. Next, the acoustic tags were mounted externally on the crabs according to the method described by [6] (Fig. 2). The tag was secured on the non-adhesive side of a Velcro strip (2.5 cm × 2.5 cm) using a cable tie and quick-setting cyanoacrylate (Pattex, super glue ultra gel). The other matching Velcro strip was attached with its non-adhesive side onto the dorsal carapace with cyanoacrylate. Finally the two adhesive sides of the Velcro strips were glued together with cyanoacrylate. Each tag was kept in place manually for 10 min. Thereafter, the crabs were placed individually in closed transparent tanks (39 × 28 × 14 cm). Each tank had a small amount of tap water that did not reach the top of the carapace, to allow the adhesives to cure. The whole tagging procedure including the drying time took about 1 h per crab. Afterwards the tanks were completely filled with tap water and aerated with an air stone. The crabs were held in the laboratory until their release on the next day (maximum 24 h after capture). This tagging technique was tested prior to the field study (Supplementary materials).

In October 2020, eight crabs were tagged and released as a first test batch (e.g. tag retention, settings tags). During this first deployment, only female crabs were used as literature suggests that only female crabs migrate through the whole estuary to release their eggs in saline water, while male adult crabs die after mating [47]. We used ID-2LP6 acoustic tags (6.3 × 22 mm, weight in air 1.9 g, weight in water 1.2 g, power output 137 db, frequency 69 kHz, ping frequency 90–150 s, estimated battery life 16.3 months) from Thelma Biotel (Norway). For this first test we chose the smallest tags available with a long battery life, since it was not sure how long the migration would take. To capture the full migration route, even if tags would detach early, mitten crabs were released at different locations within the study area (Table S1 and Fig. 1). Consequently, some individuals were released further downstream from where they were caught.

After proof of concept, the sample size was increased (following the same procedure of 2020) in the autumn of 2021 and 2022. In October and November 2021 a total of 16 crabs (equal number of males and females) were tagged. Two different tag types were used; (1) 10 crabs were tagged with the same ID-2LP6 acoustic tags as the year before and (2) 6 crabs were tagged with V9AP-2x-BLU-1 acoustic tags (9 × 35 mm, weight in air 5.3 g, weight in water 3.0 g, power output 151 db, frequency 69 kHz, ping frequency 90–150 s, estimated battery life 6.8 months) from InnovaSea (USA), equipped with both accelerometer and pressure sensor. The sensors allow to investigate activity patterns and the depth at which the crabs move. In November 2022, 10 crabs (equal number of males and females) were tagged with ID-HP9 acoustic tags (9 × 28 mm, weight in air 4.2 g, weight in water 2.4 g, power output 149 db, frequency 69 kHz, ping frequency 20–60 s, estimated battery life 6.8 months) from Thelma Biotel. We decided to use this new type of tag because we had learned that all crabs of the previous years could only be tracked for less than 6 months. We minimized the probability of not detecting a crab by increasing the power output and ping frequency at the expense of battery life beyond 6 months. All crabs tagged in 2021 and 2022 were released in the Grote Nete River at a similar distance from the estuary as their catch location (Table S1 and Fig. 1).

### Data analysis

All analyses were performed in R (version 4.2.2) and graphs were created using the package ggplot2. The significance level for statistical analyses was set at *p* < 0.05.

#### Circadian and circatidal patterns in movement

Migratory activity according to the diel and tidal cycle was analysed according to the method described by Silva et al. [60]. Arrival (first detection) and departure (last detection) of each crab at each receiver were used to narrow down migratory activity. In the case of selective migration, the crabs are assumed to arrive and depart during favourable migration conditions, such as specific tidal or circadian phases. If there is no preference for certain conditions, migratory activity is assumed to be continuous. Detections on the day of release were excluded from the analysis to avoid bias caused by the time of release.

To determine during which tidal phase (i.e. ebb and flood) arrivals and departures of crabs occurred, detections were linked to water level data from nearby tidal measuring stations. Tidal phase was determined based on the changes in water level. This approach is commonly used in other tracking studies [60, 63]. Water level data (mTAW, 10 min measuring frequency) was downloaded from waterinfo.be using the wateRinfo R package for Belgian locations and obtained from Rijkswaterstaat (waterinfo.rws.nl) for Dutch locations. The time of each detection was rounded to the nearest 10 min to match the temporal resolution of the water level data. There was generally a good overlap between the locations of the acoustic receivers and tidal measuring stations (Fig. 1). In case an acoustic receiver was located between tidal measuring stations, a weighted average of the water level data was used based on the inverse distance of the two closest tidal measuring stations. Within the tidal transition zone, only one water level measuring station was available, therefore interpolation was not possible and patterns according to tidal phase could not be analysed.

Based on the date, time and geographical position of each detection, the circadian phase (i.e. dawn, day, dusk and night) was determined (suncalc R package). Dusk was defined as the period between sunset and the end of astronomical twilight, while dawn was defined as the period between the beginning of astronomical twilight and sunrise. Due to the limited amount of observations, categories dusk and dawn were combined into one single category twilight.

In case of continuous migration, the number of arrivals or departures during each phase is expected to be proportional to the relative duration of that phase within the diel or tidal cycle [60]. When crabs selectively migrate during certain phases, observed proportions will deviate from the expected proportions. We used separate Chi-squared tests (stats R package) to test whether the observed proportions in arrivals and departures differed from expected proportions. For the analysis of the circadian pattern, this was further divided according to the tidal class (non-tidal, transition and tidal zone).

Because the relative duration of each circadian phase varies throughout the study period (winter to summer), we used a weighted average to calculate the relative duration of each phase and subsequently the expected proportions. Weights were determined based on the number of occurrences of each date within the dataset. Similarly, the relative duration of each tidal phase varies throughout the study area, therefore a weighted average was used with the number of occurrences of each location (acoustic receiver) in the dataset as weight.

#### Circadian and circatidal patterns in acceleration

Circadian and tidal phase were linked to acceleration measurements according to the method described above. We tested whether acceleration differed between circadian and tidal phases, and sex using a linear mixed effects model (‘lme’ function, nlme R package). Acceleration data was log-transformed to meet the assumptions of homogeneity of variance and normality of residuals. We created separate models for the data collected in the non-tidal, transition and tidal zone. In the model for the tidal zone (1), circadian phase, tidal phase, their interaction and sex were included as fixed factors. The models for the non-tidal and transition zone (2) included circadian phase and sex. Tidal phase was not included as a factor in the model for the transition zone due to a lack of water level data.

$$(1)\, \log \left( {acceleration} \right)\sim circadian\, phase \times tidal\, phase + sex + {(}1\, {|}\, tag\, ID/acoustic\, receiver\, ID)$$

$$(2)\,\log \left( {acceleration} \right)\sim circadian\, phase + sex + {(}1\, {|}\, tag\, ID/acoustic\, receiver\, ID)$$

We used acoustic receiver ID nested within tag ID as random factor to account for repeated measures of each crab, and an autoregressive correlation structure (‘corAR1’) to account for the correlation between acceleration measurements over time for each tag ID at each acoustic receiver. Model selection was performed based on AIC values through stepwise backward selection and the model was used as input for an ANOVA (stats R package) to test the significance of effects. ‘Emmeans’ function (emmeans R package) was used for pairwise comparisons within significant factors.

#### Migration speed

Daily migration speed (km day^−1^) was calculated for each crab between each pair of consecutive acoustic receivers by dividing the distance between those receivers by the time elapsed between the last detection at the first receiver and the first detection at the subsequent receiver. The effects of sex (fixed factor, i.e. male and female crabs), tidal class (fixed factor, i.e. non-tidal, transition and tidal zone) and their interaction on daily migration speed were tested using a linear mixed effects model (‘lmer’ function, lmerTest R package). Tag ID was included as a random effect to account for repeated measurements of the same individuals, while acoustic receiver ID was included to account for spatial variability and repeated measurements at the same locations. Daily migration speed was log-transformed to meet the assumption of homogeneity of variance. The ‘step’ function (stats R package) was used for model selection based on AIC. The model was used as input for an ANOVA (stats R package). Finally, pairwise differences (with Tukey adjustment) were tested between the different tidal classes (emmeans R package).

## Results

### Temporal and spatial scale of the migration

In total 34 Chinese mitten crabs were tagged, of which 33 were detected after release and included in the analysis, resulting in 90,107 detections, with on average (± SD) 2650 ± 2683 detections per crab (range: 16 to 10,285 detections). All crabs showed migratory behaviour, moving downstream towards the Scheldt Estuary from their release location. Mitten crabs were tracked on average (± SD) over a distance of 76 ± 40 km (range: 1 to 126 km) and a period of 109 ± 69 days (range: 2 to 301 days) (Table S2).

**Fig. 3:**
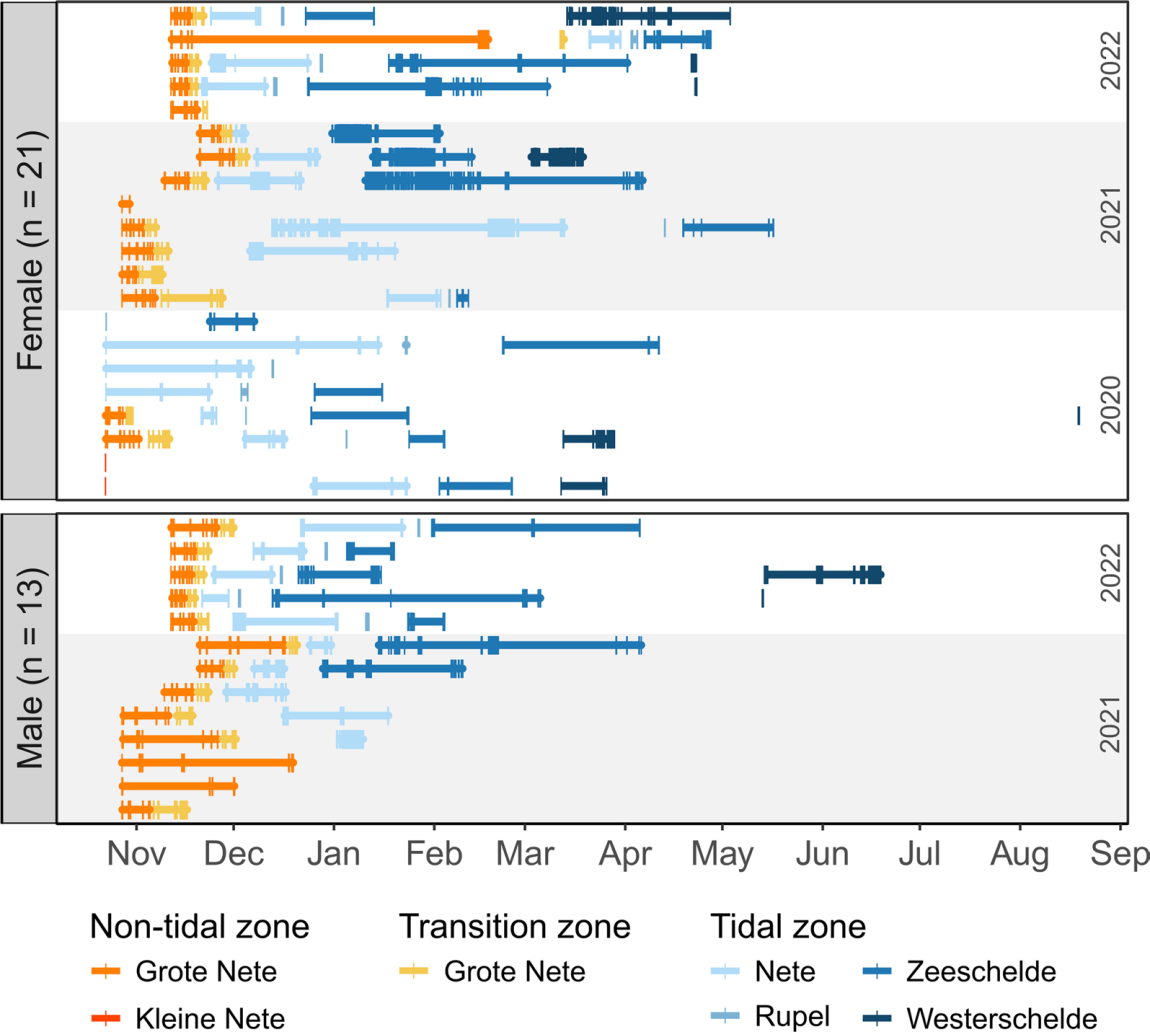
Individual trajectories of the 34 tagged Chinese mitten crabs between the period of October 2020 and July 2023. Crabs were tracked between 2 and 301 days. Vertical bars represent actual detections at receivers, while horizontal stretches indicate the period a crab was certainly present in a specific river. The year in which the individual crabs were released is depicted on the right

Over the combined years of the study, most mitten crabs were tracked as far as the Scheldt Estuary (Fig. S3), with 39% (n = 13) reaching Antwerp (Belgium, Zeeschelde) and 27% (n = 9) reaching Hansweert (the Netherlands, Westerschelde). The majority of these crabs arrived in the Scheldt Estuary during winter between mid-December and the end of January, while two crabs arrived as late as mid-April in spring (Fig. 3). The crabs arrived in the Westerschelde between the beginning of March and mid-May, with one crab being detected only in August (Fig. 3). About 18% (n = 6) of the crabs could only be tracked within River Grote Nete and are therefore considered to be lost before completing their migration. Additionally, 15% (n = 5) of the crabs reached as far as either the River Nete or River Rupel (Fig. S3). These rivers are generally not saline enough to support reproduction, leaving it uncertain whether these crabs completed their migration.

### Circadian and circatidal patterns

**Fig. 4:**
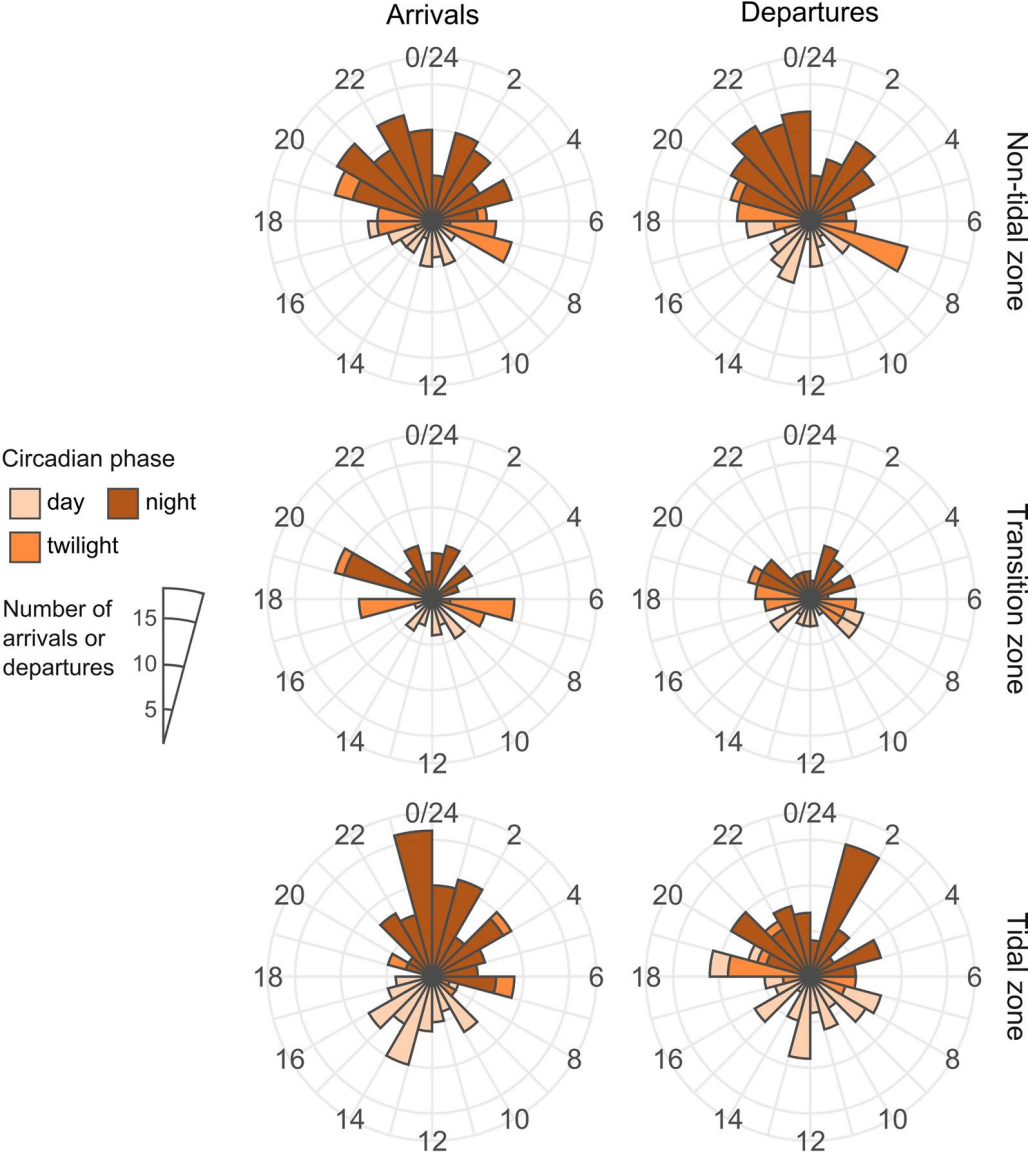
Distribution of both arrivals and departures (n = 827) of crabs at acoustic receivers in the non-tidal, transition and tidal zone, according to the diel cycle. Each radial represents 1 h. Colours visualise the time of day (circadian phase). The bars represent the number of arrivals or departures, aggregated across all receivers and crabs. The transition zone is under microtidal influence, while the tidal zone is under meso- to macrotidal influence

Analysis of arrival and departure of each individual crab at each acoustic receiver shows that in general Chinese mitten crabs migrated more during the darker hours of the day (Fig. 4) than expected in case of non-selective, continuous migration. In the non-tidal zone the observed detection proportions according to each circadian phase differed significantly from the expected proportions for both arrivals (n = 161, χ^2^ = 22.43, df = 2, *p* < 0.001) and departures (n = 162, χ^2^ = 11.39, df = 2, *p* < 0.01). Similarly, in the transition zone between the non-tidal and tidal zone, observed detection proportions differed significantly from the expected proportions for both arrivals (n = 95, χ^2^ = 8.40, df = 2, *p* < 0.05) and departures (n = 95, χ^2^ = 7.15, df = 2, *p* < 0.05), although these differences were caused by less detections during the day and more detections during twilight. In the tidal zone there was a significant difference between observed and expected proportions for arrivals (n = 157, χ^2^ = 14.42, df = 2, *p* < 0.01) but not for departures (n = 157, χ^2^ = 2.63, df = 2, *p* = 0.27).

**Fig. 5:**
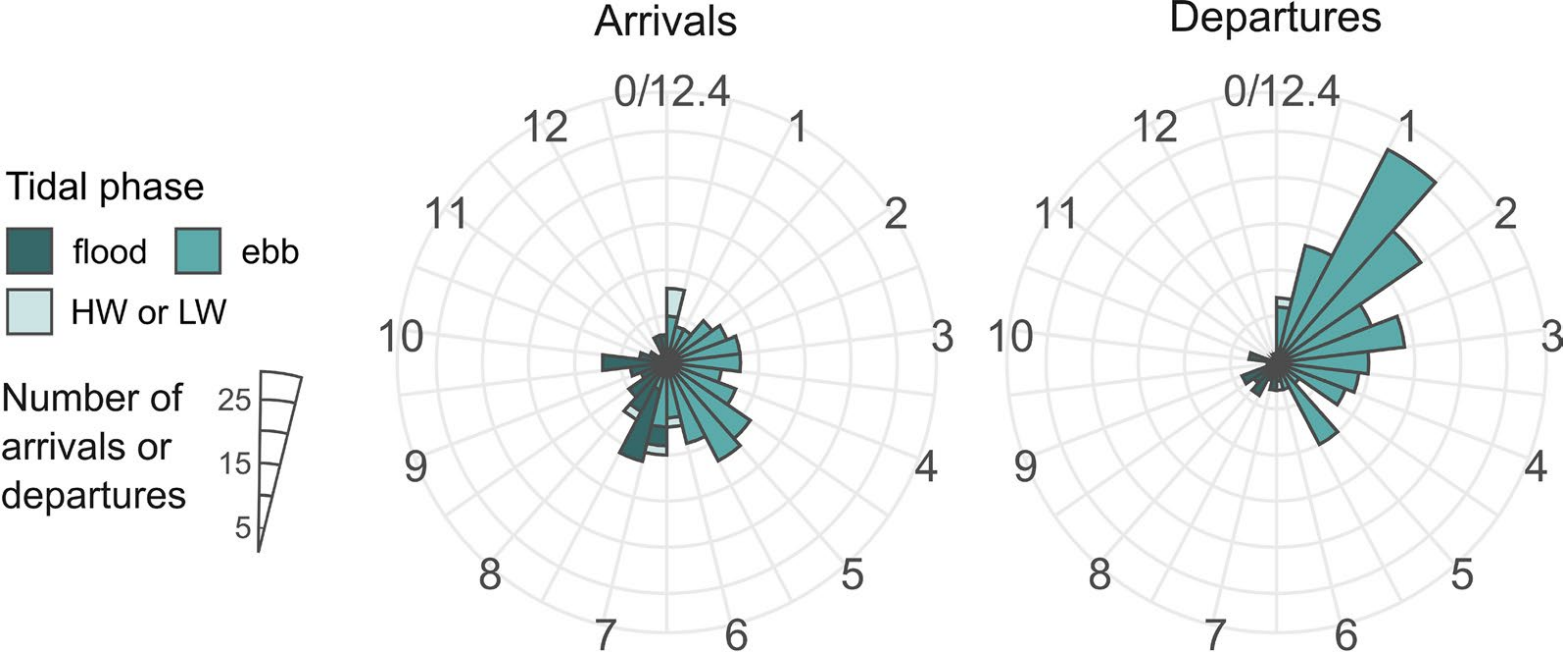
Distribution of both arrivals and departures (n = 314) of crabs at acoustic receivers in the tidal zone, according to the tidal cycle. Each radial represents 0.5 h after high water. Colours visualise the phase of the tidal cycle, with LW = low water and HW = high water. The bars represent the number of arrivals or departures, aggregated across all receivers and crabs

Based on arrivals and departures at each acoustic receiver, we found that Chinese mitten crabs migrated more during ebb (Fig. 5) than expected in case of non-selective, continuous migration. The observed detection proportions differed significantly from the expected proportions for both arrivals (n = 151, χ^2^ = 10.04, df = 1, *p* < 0.01) and departures (n = 155, χ^2^ = 60.61, df = 1, *p* < 0.001). Arrival at an acoustic receiver occurred consistently throughout the ebb phase and extended into the early part of the flood phase. Departure occurred primarily during the first half of the ebb phase.

**Fig. 6:**
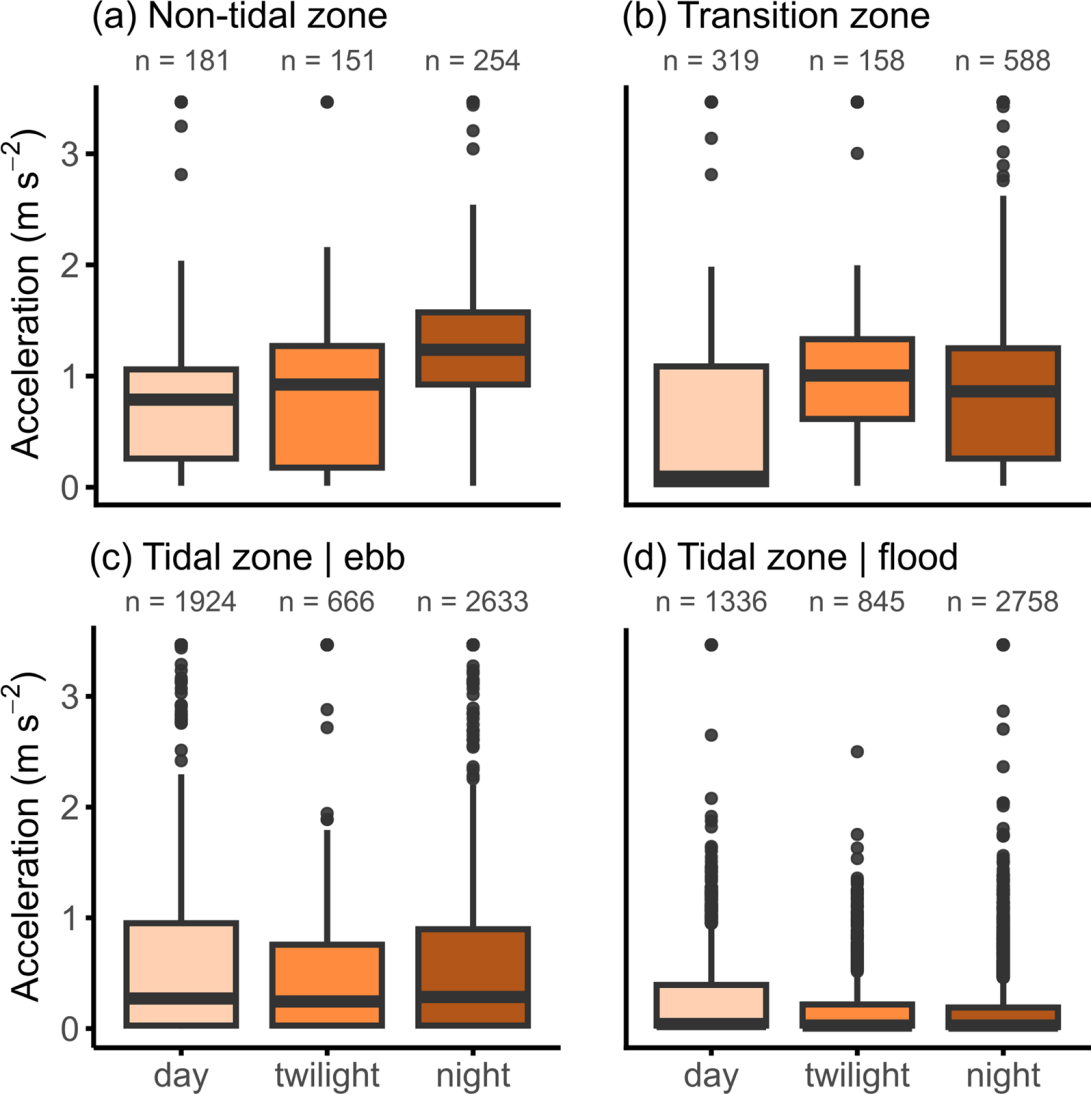
Boxplots showing the acceleration of Chinese mitten crabs (n = 6) according to circadian phase, in the non-tidal **a**, transition **b** and tidal zone **c** and **d**. The plots of the tidal zone are split up according to tidal phase. Number of observations per circadian phase and tidal class is indicated on top of each boxplot. The transition zone is under microtidal influence, while the tidal zone is under meso- to macrotidal influence

In the non-tidal and transition zone, acceleration patterns were not affected by the sex of the crab and this factor was not retained in the final model. In the non-tidal zone, the model indicated a circadian pattern (Fig. 6) in acceleration (*F*(2547) = 9.20, *p* < 0.001), with a peak in activity during the night (1.26 m s^−2^, CI_0.95_[0.92–1.72]) and reduced activity during the day (0.82 m s^−2^, CI_0.95_[0.55–1.24], *p* < 0.05) and twilight (0.62 m s^−2^, CI_0.95_[0.42–0.93], *p* < 0.001). Similarly, in the tidal transition zone, the model revealed a circadian pattern (*F*(2,1039) = 55.23, *p* < 0.001), with decreased activity during daylight hours (0.32 m s^−2^, CI_0.95_[0.17–0.60], *p* < 0.001) compared to night (1.02 m s^−2^, CI_0.95_[0.59–1.77], *p* < 0.001) and twilight (1.90 m s^−2^, CI_0.95_[1.04–3.49], *p* < 0.001).

**Fig. 7:**
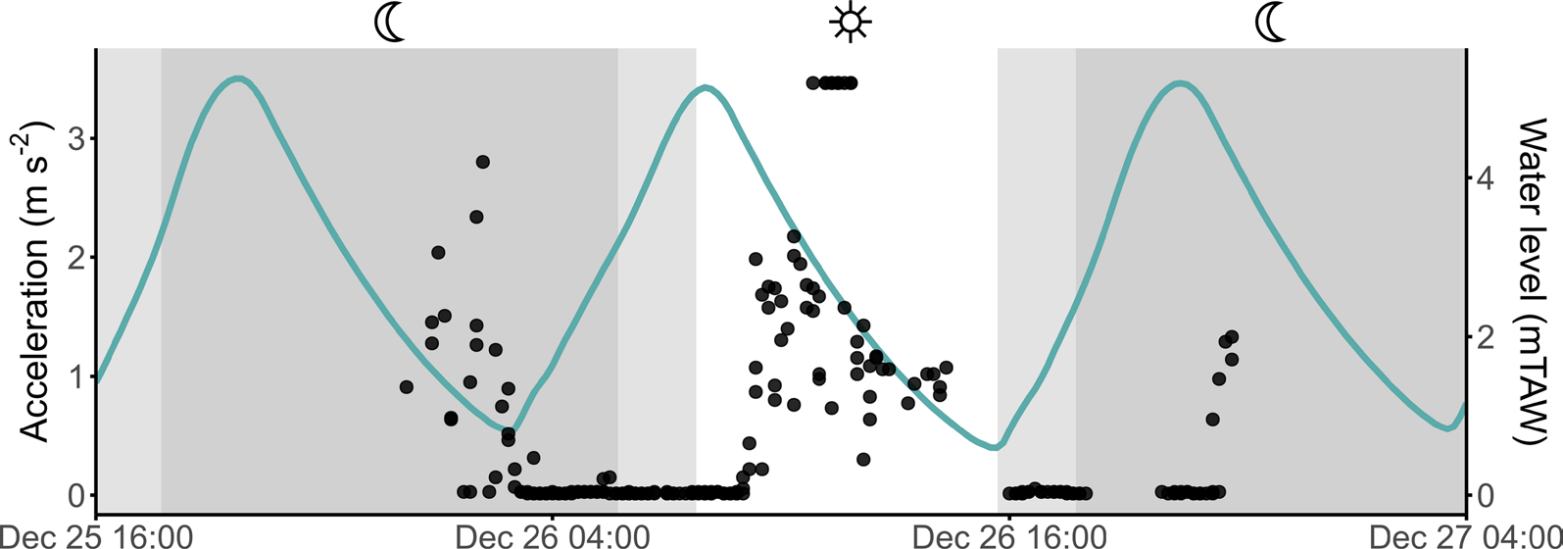
Example of acceleration patterns according to tidal and circadian phases within the tidal zone. The graph shows the data of one female crab (tag ID 3723, Table S1) at one acoustic receiver within the Nete River (tidal zone) from 25 to 27 December of 2021 (time in UTC). Acceleration (m s^−2^) measurements are shown as black dots and the blue line indicates the water level (mTAW) over time. The dark grey boxes indicate night, light grey boxes indicate twilight (i.e. dusk and dawn) and white boxes indicate day

Within the tidal zone, the model did not reveal any circadian patterns (Figs. 6 and 7) or differences between sexes in acceleration. In the final model only tidal phase was retained as a fixed factor. The model identified a pattern in acceleration according to the tidal cycle (*F*(1,10,132) = 260.54, *p* < 0.001), with an increase in crab movement during ebb (0.25 m s^−2^, CI_0.95_[0.16–0.38]), *p* < 0.001) compared to flood (0.11 m s^−2^, CI_0.95_[0.07–0.17]) (Fig. 7).

### Depth

Within the River Grote Nete, crabs migrated at an average ± SD depth of 0.97 ± 0.33 m (n = 6, range: 0.15 to 2.26 m, n observations = 661) in the non-tidal zone and 3.11 ± 1.09 m (n = 6, range: 0.45 to 6.77 m, n observations = 1072) in the transition zone. In the tidal zone, crabs were found at an average depth of 4.76 ± 1.55 m (n = 6, range: 0.30 to 8.27 m, n observations = 4135) in the Nete River, 12.29 ± 5.10 m (n = 5, range: 0.90 to 24.06 m, n observations = 5712) in the Zeeschelde, and 12.49 ± 3.45 m (n = 1, range: 2.41 to 21.96 m, n observations = 963) in the Westerschelde.

### Migration speed

**Fig. 8:**
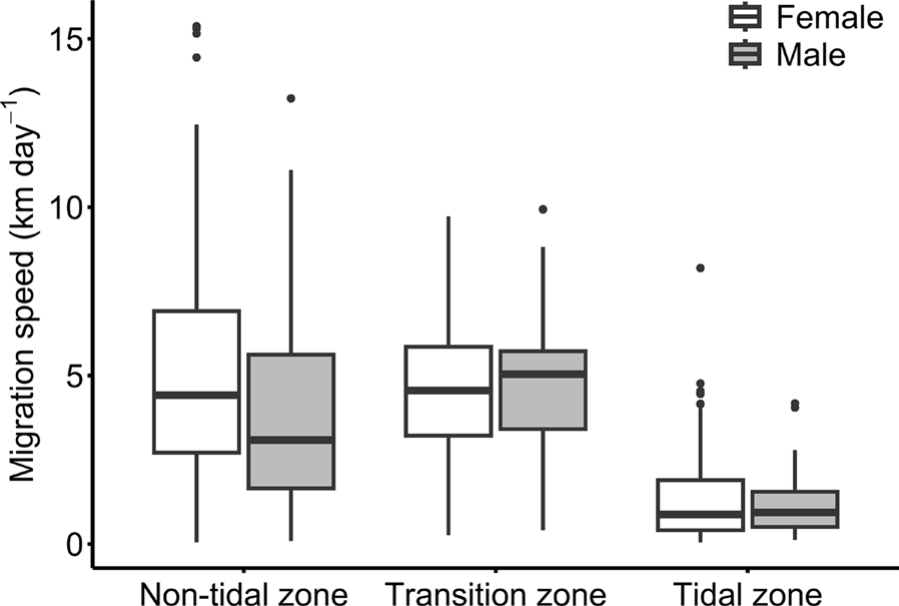
Boxplot showing the daily migration speed (km day^−1^) for female and male Chinese mitten crabs within the different tidal classes in the study area. The transition zone is under microtidal influence, while the tidal zone is under meso- to macrotidal influence

The average ± SD migration speed within the non-tidal zone was 4.65 ± 3.51 km day^−1^. However, large differences in daily migration speed were observed, ranging from 0.06 km day^−1^ up to a maximum speed of 15.37 km day^−1^ (Fig. 8). Similarly, the average ± SD migration speed within the transition zone was 4.71 ± 2.26 km day^−1^ (range 0.26 to 9.94 km day^−1^). Within the tidal rivers, the migration speed diminished and averaged ± SD at 1.29 ± 1.22 km day^−1^ (range 0.05 to 8.19 km day^−1^).

We found no significant difference between the migration speeds of male and female crabs (*F*(1, 17.21) = 0.3584, *p* = 0.557). The final model included tidal class as fixed factor and tag ID and acoustic receiver ID as random factors. Chinese mitten crabs migrated significantly (*F*(2, 21.137) = 19.19, *p* < 0.001) slower within the tidal zone (0.84 km day^−1^, CI_0.95_[0.58–1.20]) compared to the non-tidal zone (3.02 km day^−1^, CI_0.95_[2.04–4.48], *p* < 0.001) and the transition zone (3.67 km day^−1^, CI_0.95_[2.19–6.11], *p* < 0.001). We detected no significant difference in migration speed between the non-tidal, freshwater part of the study area and the transition zone (*p* = 0.80).

## Discussion

### Methodological considerations

This is the first study to apply acoustic telemetry to Chinese mitten crabs and is even one of the few applying this technique to elucidate long-distance decapod migrations. During this study, crabs were translocated from their capture site to different release locations, which may have caused short-term disorientation. However, the overall impact on behaviour is expected to be minimal. Chinese mitten crabs exhibit strong migratory instincts and, given the scale of their migration, they likely rely on general environmental cues, such as water temperature, flow direction and salinity gradients [17, 34, 55], rather than site-specific cues to guide them. Moreover, similar situations occur naturally. Crabs from isolated ponds and lakes must migrate over land to reach connected river systems [34] to continue their journey to the estuary. In these cases, the crabs also enter a new river system, where they were not resident before.

Although external attachment of tags to the carapace is relatively straightforward, tagging decapods for a longer period of time can be challenging due to moulting [18]. Mitten crabs undergo a final puberty moult before departing on their downstream spawning migration [15, 41, 47] and unlike many other crab species, mitten crabs mate when the female has a hard shell [25, 48]. The carapace width of mature adult mitten crabs can range from 30 to 95 mm [47, 55], but there is a large overlap in size with immature crabs [41]. To minimize the chance of tag loss due to moulting, large crabs (> 55 mm) captured during the autumn migration period were selected for this study, as they were assumed to have already completed their puberty moult. Despite this precaution, one out of 34 crabs was never detected, and 11 crabs were lost within the oligohaline part of the study area. Besides tag loss due to moulting, tags could have become detached through for example abrasion, although our preliminary tag retention tests did not indicate this for the applied method in controlled conditions (Supplementary materials). Furthermore, crabs might have died due to predation, environmental factors or poor health condition (e.g. infection or limited fat reserve). By selecting large individuals, we also aimed to minimize the tag burden (tag-to-body mass ratio), which ranged between 0.9 and 4.9% in this study. This is within the range that is commonly used in telemetry studies, however it is not known how tag burden affects movement rates of crabs [18, 61]. This should be further investigated in future studies to improve the application of acoustic telemetry on decapods. It is important to note that the selection of large animals may have introduced a size bias, potentially underrepresenting smaller adult crabs that might also participate in the downstream spawning migration.

In addition to tag loss or death, it is possible that crabs stopped migrating or got disoriented. Low migration speeds were commonly observed around and downstream of the city of Lier, where media articles and anecdotal observations report mitten crabs out of the water at night each year in autumn. This could signify that the combination of river confluences, old river arms within the city and sluices sometimes cause disorientation and migration delays, causing them to leave the water. Interestingly, at other confluences where acoustic receivers were present, crabs were never detected to wander in the wrong direction.

### Place of reproduction and relation to salinity

The place of reproduction can be based on the location where male Chinese mitten crabs cease their migration [49]. In this study, both male and female mitten crabs migrated until a similar distance from the estuarine mouth, although females were more frequently observed in the lower reaches of the estuary (i.e. Westerschelde). Generally, crabs migrated as far as the zone between Antwerp and Hansweert, located at 45 to 78 km from the estuarine mouth in the North Sea (Fig. S3). Although the acoustic receiver network extends further downstream of the last detections, i.e. Westerschelde and the Belgian part of the North Sea, no Chinese mitten crabs were detected in these areas. Because of this, we hypothesize that mating and spawning takes place in the zone between Antwerp and Hansweert. However, it may be possible that some crabs migrated undetected further downstream.

The zone between Antwerp and Hansweert is mesohaline and Hansweert marks the border of the polyhaline zone [44]. Salinity ranged between 0.2 and 14‰ (mean 4‰) near Antwerp and 12 and 23‰ (mean 19‰) near Hansweert, during the period that the crabs were detected (www.waterinfo.be). In the River Elbe in Germany, a salinity of 5–6‰ was suggested to be the lower limit at which mitten crabs reproduce (i.e. mating behaviour and subsequential egg-laying), which was confirmed in a laboratory study [49]. Although mating is possible at this concentration, egg attachment can be aberrant and higher concentrations of ≥ 15‰ are considered more optimal [12, 49]. Most larval stages of mitten crabs require high salinities (15–32‰), but embryos and newly hatched larvae can tolerate hyposaline conditions and have optimal survival from 5 to 20‰ [5, 12, 65], which corresponds well with the salinity range of the assumed spawning location in the Scheldt Estuary. However, the Scheldt River basin is rain fed, meaning that the salinity gradient in the estuary can change depending on the amount of freshwater discharge [44]. We therefore hypothesise that the spawning location is dynamic and can change according to the environmental circumstances.

### Timing of reproduction

In the River Grote Nete, adult mitten crabs start their downstream migration in September to late November, with a peak in October [58]. Our tracking data indicates that they arrive in brackish water between mid-December and late January. Presumably, these crabs go through their final puberty moult in August or September [33, 41, 47]. Their arrival in brackish water likely coincides with reaching sexual maturity, as the closely related Japanese mitten crab (*Eriocheir japonica*) needs four months for maturation of the ovaries after the puberty moult [41].

Once the crabs reached the assumed spawning location, they stayed around for multiple months. Generally they were detected until late April to mid-May, with two of them still being detected as late as June and August (Table S2, Fig. 2). During this time they mate and incubate their brood until larvae hatch, which may take a few months depending on environmental conditions [47, 55]. Some female crabs can even have up to three successive broods [40, 49, 55]. After the reproduction period, adult mitten crabs become inactive and are increasingly overgrown by organisms (algae, hydroids, barnacles), indicating that they lack the energy to moult [39, 47, 49]. They die soon after the reproduction period as mature adult crabs are usually found until summer [39, 40, 47, 55], which was also the case in the present study.

Overall, these results suggest that reproduction (mating, spawning and larval release) in the Scheldt Estuary takes place from at least December to June. This fits within the known time frame for reproduction of the Chinese mitten crab worldwide: in the Elbe Estuary (Germany) reproduction takes place between October and July [47], in the Tagus Estuary (Portugal) at least from March until May [4], in the Thames Estuary (United Kingdom) from September to July (Clark [10]), in San Francisco Bay (USA) between October and June [53], and in the Yangtze River (China) from October to April [33].

It is evident that in this study only a select part of the mitten crab population was tracked, possibly leading to an underestimation of the reproductive time frame. Crabs were collected from a single location within the river basin during a limited period, specifically from late October to late November. Notably, these crabs were captured approximately 140 km upstream from the river mouth, while mitten crabs can also inhabit more downstream areas [54]. Crabs that migrate earlier or reside in lower river sections may reach the estuary sooner than those monitored in this study. Conversely, it is also possible that these crabs exhibit slower migration speeds or take more breaks, resulting in similar arrival times in the estuary. Future research could further explore potential variability in migration patterns between downstream and upstream populations.

It has been suggested that some mitten crabs do not die after spawning and migrate back to upstream foraging areas [2, 34]. This hypothesis was based on the observation of a small number of crabs with barnacles attached to their carapace in inland waters [2, 34]. Furthermore, [49] proposed that at least some female crabs have the potential to spawn during a second spawning period based on histological evidence. Within the present study, we found no evidence for adult mitten crabs migrating back after reproduction. Moreover, no crabs were detected moving upstream throughout the whole study area and study period.

### Patterns in migratory behaviour

We demonstrate for the first time that Chinese mitten crabs can distinguish ebb and flood tide, as our results indicate that they selectively use the ebb tide to migrate down the estuary to reach their spawning habitat. This behaviour was earlier hypothesized by Peters and Panning [48], who suggested that mitten crabs burry themselves in the sediment during flood. While circatidal rhythms in activity are common among brachyuran crabs, typically influencing small scale movements like seeking shelter and foraging [24, 29], [30, 42, 66], there are only a few documented cases of the selective use of tides for long-distance migration. For example, adult female blue crabs (*Callinectes sapidus*) use ebb-tide transport to move to spawning locations and flood-tide transport to move back into the estuary after larval release [19, 62]. Similarly, the movement of the giant mud crab (*Scylla serrata*) is suggested to be synchronised with the tide, with female spawning migration occurring during new and full moon [23]. It is unknown how Chinese mitten crabs are able to discriminate between tides and navigate in the correct direction. This ability is likely driven by endogenous controls (e.g. circatidal or circalunidian rhythm) or exogenous cues (e.g. salinity, current velocity, hydrostatic pressure or olfactory stimuli), or a combination of both [14, 43]. As a relatively slow-moving species, the use of STST can be highly advantageous for these crabs, as it significantly reduces the energetical cost of migration, particularly in areas with high flow velocities [20].

As evidenced by our tracking and acceleration data, Chinese mitten crabs migrate mainly at night, presumably to minimize predation risks. This nocturnal migratory behaviour has also been described for adult mitten crabs in San Francisco Bay (USA) [53] and for juvenile mitten crabs in the Thames Estuary (UK) [22]. However, there is a clear change in migratory behaviour when mitten crabs enter rivers with strong tidal influence, as activity became less synchronised with circadian phases. This is in contrast to, for instance, adult female blue crabs, which primarily use nightly flood or ebb tides to move in or out the estuary during their spawning period [20]. Possibly mitten crabs predominantly act on the tidal migration cue over the diel migration cue once they are exposed to tidal currents. Their behaviour could also be influenced by increased river depth and turbidity, which reduce light levels within the tidal rivers and Scheldt Estuary compared to other rivers in the study area [1, 3, 44].

Although Chinese mitten crabs occasionally moved to shallow areas, they primarily migrated along the maximum depths of each river, which increased along their trajectory. For instance, in River Grote Nete, where the average depth is 0.94 ± 0.37 m and the maximum depth is 2.41 m (pers. comm. VMM), the crabs were typically found at an average depth of 0.97 ± 0.33 m. In the Scheldt Estuary, where reproduction takes place, they were detected at an average depth of 12.29 ± 5.10 m, while mean depth ranges between 10 and 20 m in this part of the estuary [44]. This finding is consistent with earlier reports of Chinese mitten crabs residing in the deeper parts of estuaries during winter and spring [53] and ovigerous females being collected at depths of 10–15 m in the Elbe Estuary, Germany [5]. During their migration the crabs might benefit from higher current velocities in deeper parts of the rivers compared to the shallow riverbanks. Furthermore, it is common among crustaceans to move to deeper waters during the colder months [28, 69]. While the Scheldt Estuary is well-mixed, deeper waters may still offer a more stable refuge, particularly in terms of temperature, compared to the more variable and exposed shallow areas.

### Migration speed and activity

Panning [47] derived the first migration speeds for adult mitten crabs in the 1930s, marking more than 1500 crabs in Germany during an extensive mark-recapture study. He documented migration speeds up to 8 and 12 km day^−1^ over distances of 257 to 368 km. In our study, we found that in freshwater systems with unidirectional currents, mitten crabs can reach even higher speeds of more than 15 km per day. However, generally the migration speed was lower in these rivers, averaging around 4.65 km day^−1^. Similar migration speeds in freshwater rivers have been documented in other studies. Mark-recapture studies recorded migration speeds between 0.22 and 2.78 km day^−1^ in the nearby River Kleine Nete (Belgium) [58], and between 0.3 and 5 km day^−1^ (average 1.25 km day^−1^) in freshwater channels in the Netherlands [34]. Additionally, an experimental study found an average speed of 6.4 km day^−1^ in a freshwater canal without current [17]. Differences in reported migration speeds may not only reflect population and environmental variability but could also be influenced by differences in study methodologies, such as the duration between catch checks and the spatial scale over which migration speed is measured.

Generally mitten crabs moved quickly down unidirectional freshwater rivers without interruption, which is similar to the behaviour of Japanese mitten crabs [37]. Within the transition zone to tidal rivers, where the crabs experienced limited tidal influence, the migratory behaviour and migration speed remained largely the same as in the non-tidal part. However, in the freshwater tidal parts of the study area (Rivers Nete and Rupel), a few female crabs seemed to exhibit delayed migration, staying for several months in the same area before eventually continuing moving downstream to brackish areas. Possibly these female crabs gather before moving further downstream, similar to staging behaviour of freshwater fish. Generally, in meso- and macrotidal rivers Chinese mitten crabs noticeably slowed down, with a mean migration speed of 1.29 km day^−1^. This could in part be explained by the use of STST. While this behaviour is energy efficient, it increases residence time and decreases overall migration speed because the crabs have to wait for the favourable tidal phase [60]. Additionally, there is great variability in current velocity throughout the ebbing phase, compared to the relatively stable current velocity in non-tidal rivers. Furthermore, once the mitten crabs reach suitable habitat for reproduction, migration slows down or stops, and crabs likely shift to more resident behaviour, e.g. foraging and burying in the sediment [39, 49]. For example, a tagging study on edible crabs (*Cancer pagurus*) noticed a dramatic decline in activity when females were carrying eggs [31].

## Conclusions

This study reveals key insights into the movement behaviour of the Chinese mitten crab during its downstream spawning migration. Using acoustic telemetry, we observed that mitten crabs move faster in non-tidal rivers compared to tidal rivers. In non-tidal rivers they migrate primarily during the night. In tidal rivers, migration was closely linked to the tidal cycle, with crabs taking advantage of the ebb tide to move downstream. This represents a new example of an animal using selective tidal stream transport and demonstrates that this behaviour is not only important for the larval stages of brachyuran crabs but also for adults. These findings suggest that Chinese mitten crabs adjust their movement behaviour to maximise fitness and conserve energy for spawning.

This study focused on the ecology and movement behaviour of the species. The insights of this study could also support more effective management strategies for Chinese mitten crabs. In their non-native range, removal programs may improve efficiency by targeting reproductive adults during their downstream migration, which occurs primarily at night, and by concentrating efforts in key locations where crabs take advantage of strong tidal or unidirectional currents. Conversely, in their native range, this knowledge could be applied to facilitate migration and support the continuation of their life cycle.

## Supplementary Information

Additional file 1

## Data Availability

The data are available on the European Tracking Network database: 10.14284/726.

## Abbreviation

STST: Selective tidal stream transport
