# Supplementary material for: From non-tidal to tidal environments: movement behaviour of Chinese mitten crabs on downstream spawning migration
Source: Mov Ecol. 2025 Apr 3;13:25. doi: 10.1186/s40462-025-00548-3 (PMC11969877; doi:10.1186/s40462-025-00548-3)

**Supplementary materials**

During preliminary tests, 18 adult Chinese mitten crabs (mean carapace width ± SD: 63.07 ± 5.81 mm, 4 female and 14 male) were tagged to test survival and tag retention. The dorsal carapace was dried and sanded superficially with a rotary tool. Next, a dummy tag (InnovaSea Systems Inc. V9-1x, 9 x 24 mm, weight in air 3.6 g, weight in water 2.0 g) was attached according to three different methods (six crabs per method): (1) the tag was glued to the carapace with quick-setting cyanoacrylate (Pattex, super glue ultra gel), (2) the method described in section 2.3 using a combination of cyanoacrylate and Velcro but without tie wrap, and (3) the tag was glued with a trimethoxyvinylsilane based adhesive (Tec7) on the carapace and an extra layer of adhesive was put around the tag. Each tag was kept in place manually for 10 minutes. Every crab received a unique marking with nail polish, to make sure that the individual crabs could be recognised in case a tag would fall off. Crabs were placed individually in a bucket with a small layer of tap water for 30 minutes to allow the adhesives to cure. The whole tagging procedure including the drying time took about 1 h per crab.


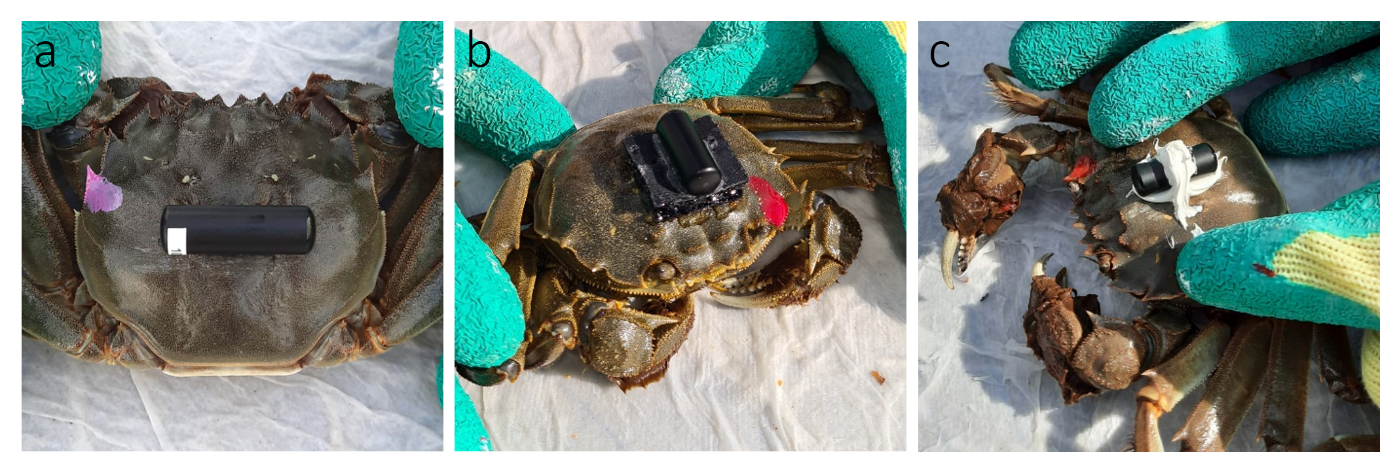


**Figure S1.** The three different adhesion methods tested: (a) the first method using cyanoacrylate, (b) the second method using a combination of cyanoacrylate and Velcro and (c) the third method using trimethoxyvinylsilane.

After tagging, crabs were kept together in three tanks (circular, 2 m diameter). Three different set-ups were used to investigate the effect of salinity and hard structures on tag retention: (1) freshwater (tap water) and no structures, (2) freshwater, sand bottom and multiple hard structures, and (3) salt water (34 ‰), sand bottom and multiple hard structures. Every tank held two crabs per adhesion method. The tanks had a water height of 1.5 m and were aerated with an air stone. The hard structures included multiple large stones, wood and hard plastic, where crabs could crawl under. The salt in the third tank was added gradually over 14 days to allow the crabs to adjust to the salinity. All crabs were fed regularly with tree leaves. The experiment lasted for more than two months.

No crabs died during the experiment. Tag retention was 67% for the first method, 83% for method two and 0% for method three. The second method gave the best results, with one tag lost in salt water after 69 days. The other tags were still firmly attached with the Velcro onto the carapace. Therefore it was decided to use this method in the field study. The method was slightly altered by adding a tie wrap to improve the attachment of the tag to the Velcro strip.


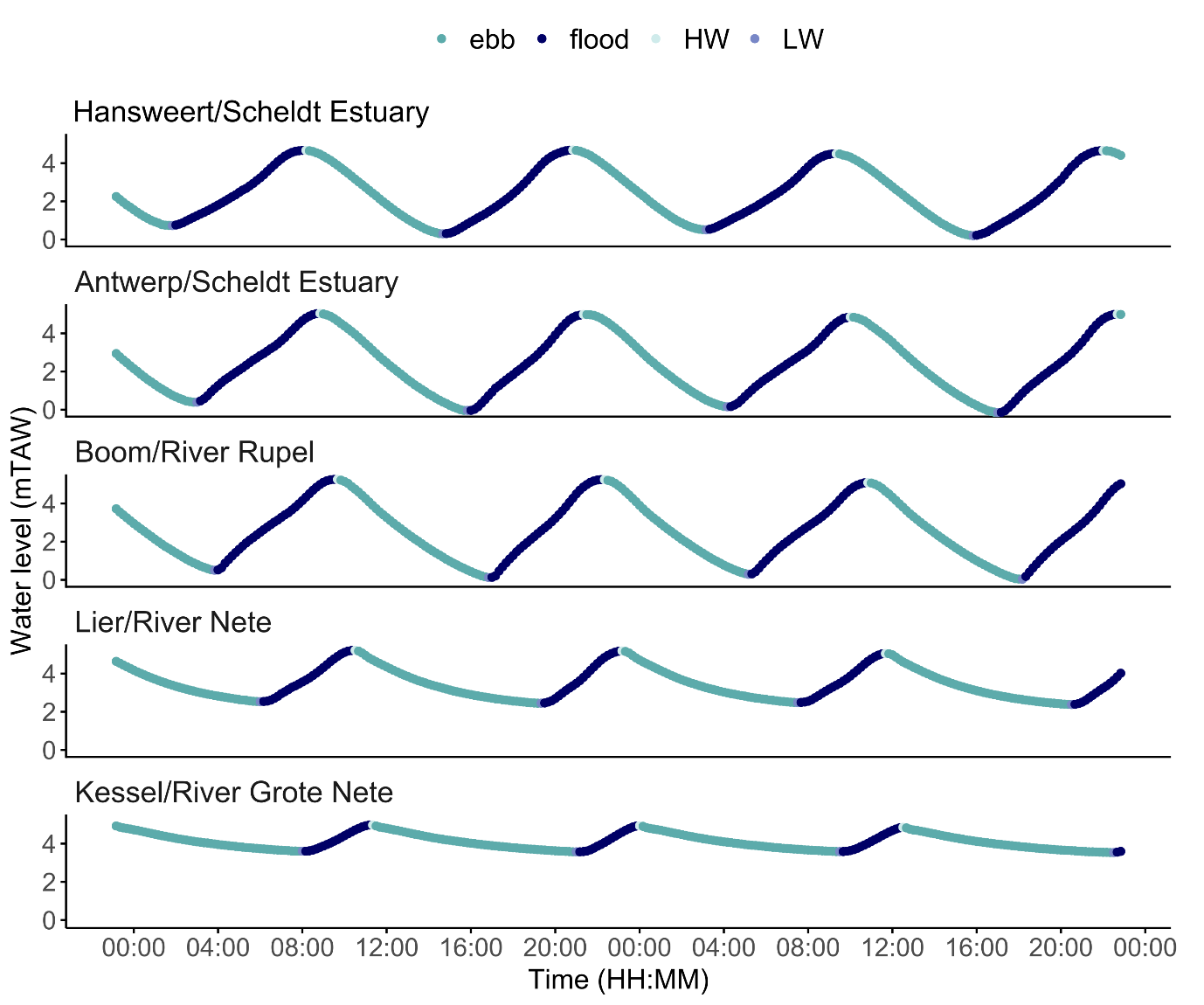
 **Figure S2**. Snapshot (2021-01-08 until 2021-01-09, hour in UTC) of water level data of five different tidal measuring stations throughout the study area shows that the duration of the tidal phases becomes increasingly asymmetrical moving landward. HW indicates high water, LW indicates low water. Note that the tidal range reaches a maximum near Antwerp.

**Table S1.** Overview of the tags, biometric measurements and release location of each Chinese mitten crab, with F = female, M = male, CW = carapace width and WW = wet weight.

| **Tag** | | **Crab** | | | | **Release** | | | |
| --- | --- | --- | --- | --- | --- | --- | --- | --- | --- |
| ID | Type | Sex | CW (mm) | | WW (g) | Date | Location | River | Coordinates |
| 9830 | ID-2LP6 | F | 64.40 | 117.87 | | 2020-10-11 | Grobbendonk | Kleine Nete | 51°11'37.3"N 4°45'14.1"E |
| 9832 | ID-2LP6 | F | 66.73 | 133.78 | | 2020-10-11 | Retie | Kleine Nete | 51°14'38.3"N 5°03'07.3"E |
| 9836 | ID-2LP6 | F | 64.18 | 119.46 | | 2020-10-11 | Geel | Grote Nete | 51°08'42.5"N 5°00'14.8"E |
| 9837 | ID-2LP6 | F | 67.47 | 141.29 | | 2020-10-11 | Zammel | Grote Nete | 51°05'28.1"N 4°56'37.9"E |
| 9840 | ID-2LP6 | F | 63.65 | 110.06 | | 2020-10-11 | Lier | Nete | 51°07'01.5"N 4°32'59.5"E |
| 9843 | ID-2LP6 | F | 63.51 | 116.88 | | 2020-10-11 | Lier | Nete | 51°07'53.4"N 4°34'52.2"E |
| 9845 | ID-2LP6 | F | 65.08 | 121.62 | | 2020-10-11 | Lier | Nete | 51°07'53.4"N 4°34'52.2"E |
| 9846 | ID-2LP6 | F | 66.97 | 133.04 | | 2020-10-11 | Niel | Rupel | 51°06'18.8"N 4°19'16.4"E |
| 9847 | ID-2LP6 | F | 59.65 | 90.26 | | 2021-10-27 | Geel | Grote Nete | 51°08'42.5"N 5°00'14.8"E |
| 9848 | ID-2LP6 | F | 62.74 | 109,69 | | 2021-10-27 | Geel | Grote Nete | 51°08'42.5"N 5°00'14.8"E |
| 9851 | ID-2LP6 | F | 61.94 | 105.71 | | 2021-10-27 | Geel | Grote Nete | 51°08'42.5"N 5°00'14.8"E |
| 9850 | ID-2LP6 | F | 61.46 | 101.57 | | 2021-10-27 | Geel | Grote Nete | 51°08'42.5"N 5°00'14.8"E |
| 9855 | ID-2LP6 | M | 73.82 | 203.6 | | 2021-10-27 | Geel | Grote Nete | 51°08'42.5"N 5°00'14.8"E |
| 9854 | ID-2LP6 | M | 63.03 | 117.57 | | 2021-10-27 | Geel | Grote Nete | 51°08'42.5"N 5°00'14.8"E |
| 9856 | ID-2LP6 | F | 64.60 | 121.76 | | 2021-10-27 | Geel | Grote Nete | 51°08'42.5"N 5°00'14.8"E |
| 9857 | ID-2LP6 | M | 62.71 | 115.54 | | 2021-10-27 | Geel | Grote Nete | 51°08'42.5"N 5°00'14.8"E |
| 9859 | ID-2LP6 | M | 62.05 | 123.69 | | 2021-10-27 | Geel | Grote Nete | 51°08'42.5"N 5°00'14.8"E |
| 9860 | ID-2LP6 | M | 58.11 | 94.62 | | 2021-10-27 | Geel | Grote Nete | 51°08'42.5"N 5°00'14.8"E |
| 3735 | V9AP-2x-BLU-1 | M | 69.22 | 170.09 | | 2021-11-09 | Geel | Grote Nete | 51°08'17.4"N 4°59'46.5"E |
| 3733 | V9AP-2x-BLU-1 | F | 62.89 | 108.89 | | 2021-11-09 | Geel | Grote Nete | 51°08'17.4"N 4°59'46.5"E |
| 3723 | V9AP-2x-BLU-1 | F | 66.03 | 126.63 | | 2021-11-20 | Geel | Grote Nete | 51°08'17.4"N 4°59'46.5"E |
| 3725 | V9AP-2x-BLU-1 | M | 62.21 | 117.25 | | 2021-11-20 | Geel | Grote Nete | 51°08'17.4"N 4°59'46.5"E |
| 3727 | V9AP-2x-BLU-1 | M | 68.95 | 151.86 | | 2021-11-20 | Geel | Grote Nete | 51°08'17.4"N 4°59'46.5"E |
| 3729 | V9AP-2x-BLU-1 | F | 64.81 | 114.98 | | 2021-11-20 | Geel | Grote Nete | 51°08'17.4"N 4°59'46.5"E |
| 6925 | ID-HP9 | M | 68.15 | 155.55 | | 2022-11-11 | Geel | Grote Nete | 51°08'17.4"N 4°59'46.5"E |
| 6926 | ID-HP9 | F | 67.67 | 134.44 | | 2022-11-11 | Geel | Grote Nete | 51°08'17.4"N 4°59'46.5"E |
| 6927 | ID-HP9 | M | 78.08 | 228.32 | | 2022-11-11 | Geel | Grote Nete | 51°08'17.4"N 4°59'46.5"E |
| 6928 | ID-HP9 | M | 70.22 | 171.88 | | 2022-11-11 | Geel | Grote Nete | 51°08'17.4"N 4°59'46.5"E |
| 6929 | ID-HP9 | F | 65.06 | 117.44 | | 2022-11-11 | Geel | Grote Nete | 51°08'17.4"N 4°59'46.5"E |
| 6930 | ID-HP9 | M | 69.62 | 149.92 | | 2022-11-11 | Geel | Grote Nete | 51°08'17.4"N 4°59'46.5"E |
| 6931 | ID-HP9 | F | 66.96 | 126.83 | | 2022-11-11 | Geel | Grote Nete | 51°08'17.4"N 4°59'46.5"E |
| 6932 | ID-HP9 | F | 65.34 | 118.02 | | 2022-11-11 | Geel | Grote Nete | 51°08'17.4"N 4°59'46.5"E |
| 6933 | ID-HP9 | F | 64.16 | 117.49 | | 2022-11-11 | Geel | Grote Nete | 51°08'17.4"N 4°59'46.5"E |
| 6934 | ID-HP9 | M | 65.84 | 128.75 | | 2022-11-11 | Geel | Grote Nete | 51°08'17.4"N 4°59'46.5"E |

**Table S2.** Overview of total distance, time and detections of each tagged Chinese mitten crab, with F = female and M = male, total time in days, and total distance in km.

| **Tag ID** | **Sex** | | **First detection** | | **Last detection** | | **Total time** | **Total detections** | **Total distance** |
| --- | --- | --- | --- | --- | --- | --- | --- | --- | --- |
| 9830 | F | | 2020-10-22 | | 2021-03-26 | | 155 | 482 | 101 |
| 9832 | F | | 2020-10-22 | | 2020-10-22 | | NA | 0 | NA |
| 9836 | F | | 2020-10-22 | | 2021-03-28 | | 157 | 1964 | 126 |
| 9837 | F | | 2020-10-22 | | 2021-08-19 | | 301 | 362 | 117 |
| 9840 | F | | 2020-10-22 | | 2021-01-15 | | 85 | 110 | 40 |
| 9843 | F | | 2020-10-22 | | 2020-12-13 | | 52 | 269 | 19 |
| 9845 | F | | 2020-10-22 | | 2021-04-11 | | 171 | 542 | 61 |
| 9846 | F | | 2020-10-22 | | 2020-12-07 | | 46 | 32 | 19 |
| 9847 | F | | 2021-10-27 | | 2022-02-11 | | 107 | 1006 | 86 |
| 9848 | F | | 2021-10-27 | | 2021-11-09 | | 13 | 1166 | 42 |
| 9850 | F | | 2021-10-27 | | 2022-01-19 | | 84 | 1445 | 55 |
| 9851 | F | | 2021-10-27 | | 2022-05-16 | | 201 | 2958 | 100 |
| 9854 | M | | 2021-10-27 | | 2021-11-16 | | 20 | 1443 | 42 |
| 9855 | M | | 2021-10-27 | | 2021-12-01 | | 35 | 110 | 12 |
| 9856 | F | | 2021-10-27 | | 2021-10-29 | | 2 | 16 | 1 |
| 9857 | M | | 2021-10-27 | | 2021-12-19 | | 53 | 210 | 12 |
| 9859 | M | | 2021-10-27 | | 2022-01-10 | | 75 | 1910 | 46 |
| 9860 | M | | 2021-10-27 | | 2022-01-17 | | 82 | 1083 | 55 |
| 3733 | F | | 2021-11-09 | | 2022-04-06 | | 148 | 7366 | 99 |
| 3735 | M | | 2021-11-09 | | 2021-12-17 | | 38 | 1986 | 54 |
| 3723 | F | | 2021-11-20 | | 2022-03-18 | | 118 | 5971 | 124 |
| 3725 | M | | 2021-11-20 | | 2022-02-09 | | 81 | 3382 | 99 |
| 3727 | M | | 2021-11-20 | | 2022-04-05 | | 136 | 2543 | 99 |
| 3729 | F | | 2021-11-20 | | 2022-02-02 | | 74 | 3204 | 85 |
| 6925 | M | | 2022-11-11 | | 2023-02-04 | | 85 | 6877 | 85 |
| 6926 | F | | 2022-11-11 | | 2022-11-22 | | 11 | 443 | 35 |
| 6927 | M | | 2022-11-11 | | 2023-05-13 | | 183 | 2002 | 126 |
| 6928 | M | | 2022-11-11 | | 2023-06-18 | | 219 | 5574 | 124 |
| 6929 | F | | 2022-11-11 | | 2023-04-23 | | 162 | 4960 | 124 |
| 6930 | M | | 2022-11-11 | | 2023-01-19 | | 69 | 4042 | 85 |
| 6931 | F | | 2022-11-11 | | 2023-04-22 | | 162 | 10285 | 125 |
| 6932 | F | | 2022-11-11 | | 2023-04-27 | | 167 | 8093 | 85 |
| 6933 | F | | 2022-11-11 | | 2023-05-03 | | 173 | 3537 | 124 |
| 6934 | M | | 2022-11-11 | | 2023-04-05 | | 145 | 4734 | 99 |
|  | |  | |  | | **Average ± SD** | 109 ± 69 | 2731 ± 2684 | 76 ± 40 |

**Figure S3**. Map of the study area showing the migration endpoints of tagged Chinese mitten crabs (indicated in red). Of the tracked crabs, 18% (n = 6) were last detected within the River Grote Nete, and 15% (n = 5) within the Rivers Nete or Rupel. None (0%) were lost between the confluence of the Rivers Scheldt and Rupel and the city of Antwerp. A total of 39% (n = 13) were last detected near Antwerp, while 27% (n = 9) were last recorded near Hansweert, both locations within the Scheldt Estuary.


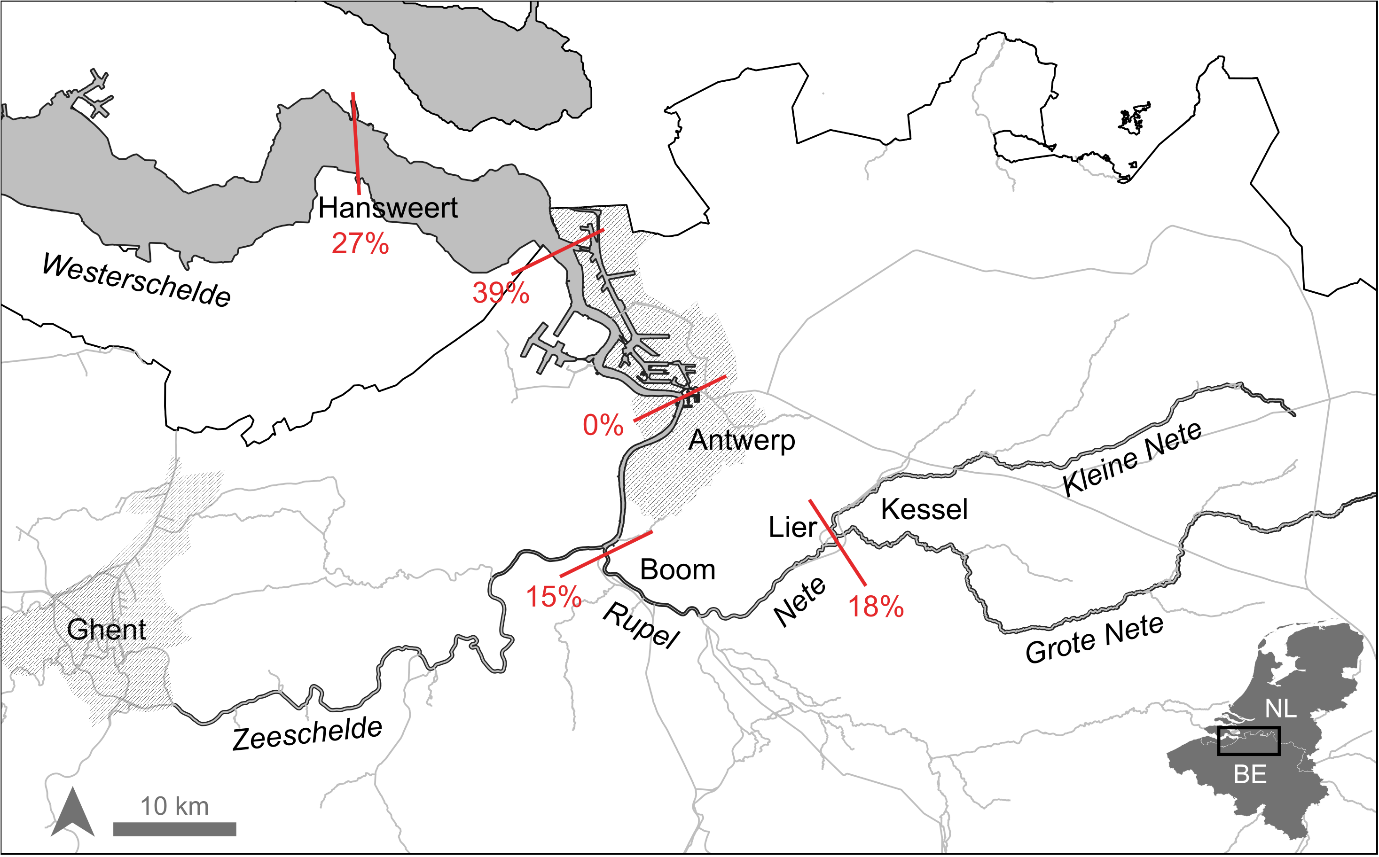

Supplement: Supplementary file 1 — Additional file 1 [file 40462_2025_548_MOESM1_ESM.docx]
